# Lack of Direct Effects of Neurotrophic Factors in an In Vitro Model of Neuroinflammation

**DOI:** 10.3390/ijms25084160

**Published:** 2024-04-09

**Authors:** Nimra Aziz, Chiara Ruzza, Chiara Falcicchia, Annunziata Guarino, Marie Soukupova, Laila Asth, Valentina Aleotti, Barbara Bettegazzi, Michele Simonato, Silvia Zucchini

**Affiliations:** 1 Department of Neuroscience and Rehabilitation, University of Ferrara, via Fossato di Mortara 70, 44121 Ferrara, Italy; nimra.aziz@unife.it (N.A.); grnnnz@unife.it (A.G.); marie.soukupova@unife.it (M.S.); dsllla@unife.it (L.A.); smm@unife.it (M.S.); zcs@unife.it (S.Z.); 2 Laboratory of Technologies for Advanced Therapy (LTTA), Technopole of Ferrara, 44121 Ferrara, Italy; 3 CNR Institute of Neuroscience, 56124 Pisa, Italy; chiara.falcicchia@unife.it; 4 Operating Unit Neurological Clinic, University Hospital of Ferrara, via Aldo Moro 8, 44124 Ferrara, Italy; valentina.aleotti@unife.it; 5 School of Medicine, University Vita-Salute San Raffaele, via Olgettina 58, 20132 Milan, Italy; bettegazzi.barbara@hsr.it; 6 Division of Neuroscience, IRCCS San Raffaele Scientific Institute, Via Olgettina 60, 20132 Milan, Italy

**Keywords:** neuroinflammation, brain-derived neurotropic factor, fibroblast growth factor-2, cytokines, lipopolysaccharide

## Abstract

Neuroinflammation is associated with several neurological disorders including temporal lobe epilepsy. Seizures themselves can induce neuroinflammation. In an in vivo model of epilepsy, the supplementation of brain-derived neurotropic factor (BDNF) and fibroblast growth factor-2 (FGF-2) using a Herpes-based vector reduced epileptogenesis-associated neuroinflammation. The aim of this study was to test whether the attenuation of the neuroinflammation obtained in vivo with BDNF and FGF-2 was direct or secondary to other effects, for example, the reduction in the severity and frequency of spontaneous recurrent seizures. An in vitro model of neuroinflammation induced by lipopolysaccharide (LPS, 100 ng/mL) in a mouse primary mixed glial culture was used. The releases of cytokines and NO were analyzed via ELISA and Griess assay, respectively. The effects of LPS and neurotrophic factors on cell viability were determined by performing an MTT assay. BDNF and FGF-2 were tested alone and co-administered. LPS induced a significant increase in pro-inflammatory cytokines (IL-1β, IL-6, and TNF-α) and NO. BDNF, FGF-2, and their co-administration did not counteract these LPS effects. Our study suggests that the anti-inflammatory effect of BDNF and FGF-2 in vivo in the epilepsy model was indirect and likely due to a reduction in seizure frequency and severity.

## 1. Introduction

Neuroinflammation is a well-orchestrated and complex process involving glial cells, particularly astrocytes and microglia. Many lines of evidence have revealed the association of neuroinflammation with neurological diseases including epilepsy [1,2]. Experimental data have demonstrated that acute seizures can induce neuroinflammation and that spontaneous recurrent seizures (SRSs) can cause chronic neuroinflammation [3]. On the other hand, a persistent and sustained neuroinflammation can facilitate the occurrence of seizures through the continuous release of inflammatory cytokines and reactive oxygen species (ROS), impairing neuronal plasticity and leading to cell death [4].

Understanding the molecular mechanisms that contribute to epileptogenesis may pave the way for the development of new therapeutic targets. In this regard, neurotrophic factors (NTFs) represent widely studied molecules given the extensive research evidence indicating their involvement in many of the cellular changes associated with epilepsy [5]. NTFs are indeed involved in the growth, survival, and differentiation of developing and mature neurons. In the adult central nervous system (CNS), they exert not only these neurotrophic effects but also functional effects at the synaptic level [6]. These features have led to the hypothesis that some NTFs, in particular the fibroblast growth factor-2 (FGF-2) and the brain-derived neurotrophic factor (BDNF), may positively impact epileptogenesis-associated cell death and circuit rearrangements [7]. In fact, both FGF-2 and BDNF protect neurons from damage and, furthermore, FGF-2 is a potent proliferation factor for neural stem cells [8] whereas BDNF favors their differentiation into neurons [9].

In fact, the local delivery (achieved using a non-replicating herpes-virus-based vector) of a combination of FGF-2 and BDNF during epileptogenesis was found to limit damage, favor the proliferation of hippocampal neural stem cells and their differentiation into neurons, and prevent SRSs [10]. In addition, it was found that this treatment also produced robust anti-inflammatory effects. Specifically, the delivery of FGF-2 and BDNF in the rat hippocampus after pilocarpine-induced status epilepticus reduced various markers of inflammation including astrocytosis, microgliosis, and IL-1β expression. In particular, IL-1β expression was almost completely prevented by the supplementation of these NTFs [11].

This work focused on the question of whether the anti-inflammatory effect of FGF-2 and BDNF was directly produced by these NTFs or was secondary to other effects, for example, the reduction in SRS severity and frequency. We aimed to address this question by directly applying FGF-2 and BDNF in an in vitro model of neuroinflammation, the primary mixed glial cell (MGC) culture treated with bacterial endotoxin lipopolysaccharides (LPSs). Activated glial cells are a crucial source of pro-inflammatory mediators such as cytokines, chemokines, and nitric oxide (NO) [12,13,14]. MGC cultures are highly sensitive to LPS and provide a good model for studying neuroinflammation. Of course, this may be viewed as a simplistic model of neuroinflammation at large, not at all specific for epilepsy.

## 2. Results

### 2.1. Characterization of the Primary Mixed Glial Culture

**Figure 1:**
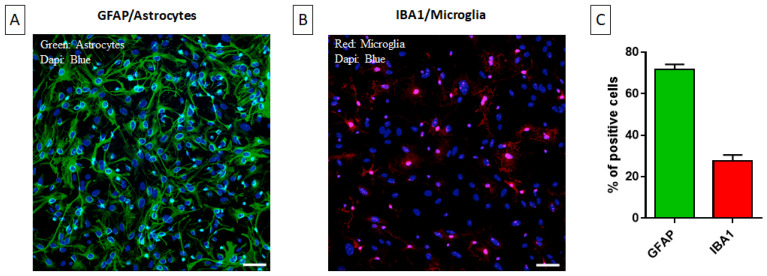
Astrocytes and microglia in MGC culture as measured through immunofluorescence using (**A**) GFAP (green; astrocytes) and (**B**) Iba1 (red; microglia) antibodies. Nuclei were stained using DAPI (blue). Scale bar = 200 μm. (**C**) Percentage of putative astrocytes (GFAP-positive cells) and putative microglial cells (IBA1-positive) cells in MGC culture. Data are reported as percents of DAPI-positive cells. Quantification was performed using the ImageJ 1.53k software. Results are the means ± SEMs of three independent experiments. Abbreviations: GFAP = Glial fibrillary acidic protein (GFAP); IBA1 = Ionized calcium binding adaptor molecule 1; DAPI = 4′,6-diamidino-2-phenylindole.

Astrocytes and microglial cells are engaged constantly in a fine crosstalk in the frame of neuroinflammatory events [15]. As both microglia and astrocytes are involved in neuroinflammation, we decided to use MGCs for our experiments. Immunofluorescence was applied for the determination of the relative representation of astrocytes and microglia using GFAP (astrocyte-specific) and Iba1 (microglia-specific) antibodies (Figure 1A,B). MGCs were composed of 70–75% astrocytes and 20–25% microglia (Figure 1C).

### 2.2. Setting up a Neuroinflammation In Vitro Model: Concentration- and Time-Dependent Effect of LPS on Cell Viability, Cytokine Level, and NO Production

**Figure 2:**
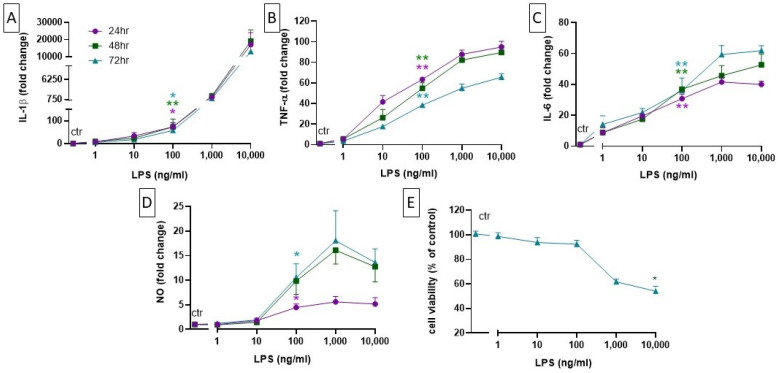
Concentration-dependent effect of LPS on the release of IL-1β (**A**), TNF-α (**B**), IL-6 (**C**), and NO (**D**) from MGC culture after 24 (purple), 48 (green), and 72 h (blue) of treatment. The release of cytokines was evaluated using a commercially available ELISA plate (Ella). NO release was determined using the Griess assay and readings of absorbance were made at 570 nm using a multimode plate reader. (**E**) Cell viability was assessed at 72 h using the MTT assay and reading absorbance at 550 nm. Data are presented as the means ± SEMs of three independent experiments run in duplicate. Statistical analysis was performed using Kruskal–Wallis one-way ANOVA followed by the Dunn’s test. * *p* < 0.05; ** *p* < 0.01 against control. Abbreviations: LPS = lipopolysaccharide; IL-1β = interleukin-1 beta; TNF-α = tumor necrosis factor-alpha; IL-6 = interleukin-6.

Both astrocytes and microglial cells, when acquiring an activated state in response to potentially noxious insults, can release pro-inflammatory molecules like TNF-α, IL-6, IL-1β, and NO [16]. LPS has been used to mimic the inflammatory process, stimulating cells to release pro-inflammatory cytokines. In order to choose an optimal LPS concentration, we first investigated cell viability after 72 h of exposure by using the MTT assay. We observed that only the highest concentration of LPS (10,000 ng/mL) significantly reduced cell viability, but a tendency to reduction could also be observed at 1000 ng/mL while no death was detected at up to 100 ng/mL (Figure 2E).

We then measured the LPS-induced release of cytokines at different time points. Cells were incubated with different concentrations of LPS (1, 10, 100, 1000, 10,000 ng/mL); their supernatants were collected at 24, 48, and 72 h; and the release of cytokines was initially measured using a multiplex assay (MILLIPLEX MAP, Merck). While we could not detect the release of cytokines from primary neuronal cultures, we observed a strong, LPS-concentration-dependent increase in the levels of selected cytokines (IL-1β, TNF-α, and IL-6) in the MGC culture. Precise levels of release of these cytokines were therefore measured using ELISA (Figure 2A–C). Of note, LPS 100 ng/mL, under these experimental conditions, did not induce the release of the anti-inflammatory cytokines IL-4 and IL-10.

In addition to cytokines, microglial cells release reactive oxygen species (ROS) in response to noxious stimuli. ROS increase the expression of the inducible nitric oxide synthase (iNOS) and thereby promote an excessive production of NO [17]. The production of NO from microglia stimulates the activation of astrocytes, which then further enhance NO production [18]. To determine the occurrence of these phenomena in our system, cells were treated with different LPS concentrations (1–10,000 ng/mL) for 72 h and NO production was analyzed indirectly via nitrite concentration measurement in the cell supernatant at different time points. As expected, we observed a significant, LPS-concentration- and time-dependent increase in NO production (Figure 2D). Based on the results obtained with the MTT assay, in the cytokines and NO release experiments, the LPS doses of 1000 and 10,000 ng/mL were excluded from the statistical analysis to avoid possible confounding effects due to cell death.

Taken together, these results suggest that 100 ng/mL is the lowest effective concentration of LPS able to increase the production of cytokines and NO without affecting the viability of an MGC culture. Thus, we decided to use this concentration in all subsequent experiments.

### 2.3. Effects of FGF-2 and BDNF on Cell Viability, Cytokine Level, and NO Production

**Figure 3:**
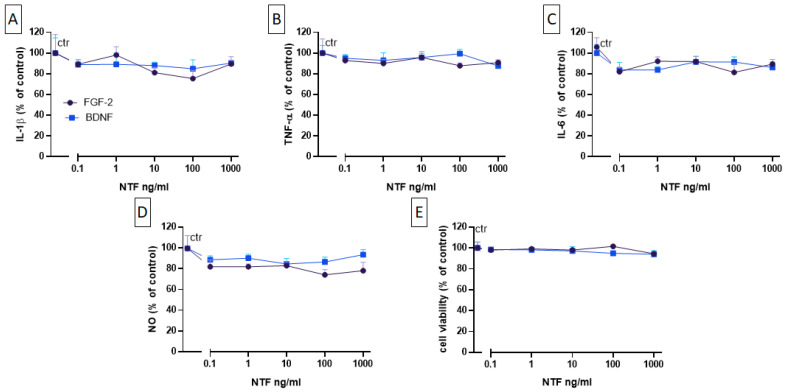
Effects of FGF-2 (purple) and BDNF (blue) on the release of IL-1β (**A**), TNF-α (**B**), IL-6 (**C**), and NO (**D**) from 100 ng/mL LPS-stimulated MGCs. Cells were treated with LPS for 24 h and then with FGF-2 or BDNF for 48 h. Releases of cytokines and NO were evaluated as described in Figure 2. (**E**) Effects of FGF-2 and BDNF on cell viability were measured using the MTT assay. Data are presented as the means ± SEMs of five independent experiments run in duplicate. Statistical analysis was performed using Kruskal–Wallis one-way ANOVA followed by Dunn’s test against the LPS-treated control. Abbreviations: FGF-2 = fibroblast growth factor-2; BDNF = brain-derived neurotrophic factor; LPS = lipopolysaccharide; IL-1β = interleukin-1 beta; TNF-α = tumor necrosis factor-alpha; IL-6 = interleukin-6.

Next, we examined the effects of BDNF and FGF-2 on cytokines and NO LPS-induced levels. MGCs were incubated with different concentrations of FGF-2 and BDNF (0.1, 1, 10, 100, 1000 ng/mL) for 48 h following 24 h exposure to vehicle (i.e., basal conditions) or to LPS (100 ng/mL). Under basal conditions, FGF-2 and BDNF did not modify the release of IL-1β, TNF-α, and IL-6. Both NTF factors had no effects also on LPS-induced cytokines and on NO levels (Figure 3A–D), as well as on cell viability (Figure 3E). Additionally, both NTFs did not modify the levels of the anti-inflammatory factors IL-4 and IL-10, neither alone nor in the presence of LPS.

### 2.4. Effects of FGF-2 and BDNF Co-Administration on Cell Viability, Cytokine Level, and NO Production

**Figure 4:**
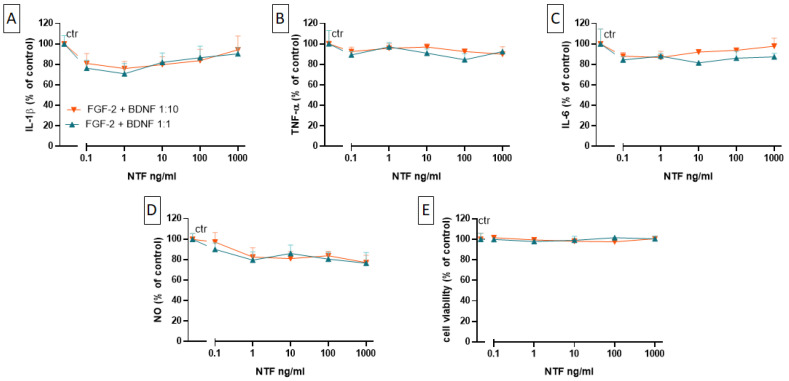
Effects of co-administration of FGF-2 and BDNF at 1:1 (blue) and 1:10 ratios (orange) on the release of IL-1β (**A**), TNF-α (**B**), IL-6 (**C**), and NO (**D**) from 100 ng/mL LPS-stimulated MGCs. Cells were treated with LPS (100 ng/mL) for 24 h and then with FGF-2/BDNF for 48 h. Releases of cytokines and NO were evaluated as described in Figure 2. (**E**) Effects of FGF-2 and BDNF on cell viability were measured using the MTT assay. Data are presented as the means ± SEMs of five independent experiments run in duplicate. Statistical analysis was performed using Kruskal–Wallis one-way ANOVA followed by Dunn’s test against the LPS-treated control. Abbreviations: FGF-2 = fibroblast growth factor-2; BDNF = brain-derived neurotrophic factor; LPS = lipopolysaccharide; IL-1β = interleukin-1 beta; TNF-α = tumor necrosis factor-alpha; IL-6 = interleukin-6.

Although no reduction in cytokine levels and NO production could be observed after the administration of BDNF or FGF-2 alone, in line with data published by [11], we decided to co-administer BDNF and FGF-2. The FGF-2 and BDNF co-administration effects were determined following two different concentration ratios: one 1:1 FGF-2:BDNF but also a 1:10 FGF-2:BDNF, chosen considering the fact that BDNF is about 10 times more potent than FGF-2 [14,19,20,21]. However, neither co-administration ratio had any effect on LPS-induced cytokine and NO levels (Figure 4A–D). No effect was observed on cell viability (Figure 4E). Also, no effect was detected on IL-4 and IL-10 release.

## 3. Discussion

In this study, we found that FGF-2 and BDNF, both individually and in combination, are unable to modulate the release by glia of neuroinflammatory factors, such as pro-inflammatory cytokines and nitric oxide (NO), in an in vitro model of neuroinflammation, indicating that their effects in intact animals are likely indirect. First, we decided to use a primary culture of mixed glia as a model that allows one to mimic the physiopathological activation and crosstalk between microglia and astrocytes in a controlled environment. Given the high reactivity of glial cells, it is possible that different preparation methods result in different phenotypes and compositions, which in turn may affect experimental replicates. In our hands, however, the cellular composition was constant across different experiments. Primary mixed cell cultures have, indeed, the ability to reproduce various established physiological properties of microglia such as migration, the ingestion of debris, and the secretion of pro-inflammatory cytokines and chemokines upon activation [22,23]; microglial cells closely cooperate with astrocytes, which play an essential role in maintaining the local physiological microenvironment by controlling potassium and neurotransmitter levels, thereby exerting a profound influence on neuronal excitability [24].

Here, we used a model of the LPS-induced release of inflammation mediators in these cultures. LPS initiates the process of neuroinflammation by binding to the toll-like receptor-4 (TLR4) and activating downstream signaling pathways resulting in the production of cytokines and chemokines including TNF-α, IL-6, IL-1β, and NO [18,25]. In vitro models, like that of LPS-induced inflammation, are useful to reproduce aspects of the interactions that occur in the brain. Altogether, neurons, astrocytes, and microglia play a pivotal role in shaping neuroinflammation as a response to insults in the CNS. Although the LPS model presents some limitations, since it cannot fully mimic epileptogenesis-associated neuroinflammation, there is evidence demonstrating that the acute administration of LPS induces the release of TNF-α and IL-1β and facilitates epileptiform discharges in brain slices in vitro [26]. These effects seem to depend on increased excitatory synaptic strength, because an enhancement of excitatory post-synaptic currents (EPSCs), but not of inhibitory post-synaptic currents (IPSCs), has been observed in hippocampal slices [26]. This is in line with other studies reporting that LPS induces glutamate release [27] and that this increased release may originate from glial, rather than neuronal, elements in cortical slices [28].

In this study, we aimed to focus on neuroinflammation because of the accumulation of evidence that, together with oxidative stress, this is one of the most important processes causing neuronal cell death and prompting epileptogenesis [29]. Astrocytes and microglial cells are engaged constantly in a fine crosstalk during the neuroinflammation process: reactive microglial cells release pro-inflammatory cytokines and NO-activating astrocytes, which switch to an astrogliosis state and ultimately secrete more pro-inflammatory molecules, initiating a feed-forward loop [16,30]. Hence, we decided to optimize our model by using a mixed culture of astrocytes and microglia and LPS as an inducer of neuroinflammation.

BDNF and FGF-2 treatments have been reported to reduce neuroinflammation in rats injected with the pro-epileptic drug, pilocarpine [11], and in other disease models [31,32]. In fact, the concomitant overexpression of both NTFs in the hippocampus in pilocarpine rats, in addition to improving the epileptic outcome (i.e., reducing frequency and severity of spontaneous recurrent seizures), has also reduced neuroinflammatory-related parameters. In a study, BDNF and FGF-2 induced a rapid attenuation of astrocytosis and microgliosis and prevented the IL-1β overexpression that is typical of the post-status epilepticus period [11]. Neuroinflammation has been implicated in the generation of seizures and in the progression to epilepsy both in preclinical [33,34] and clinical studies [35]. Thus, it is tempting to hypothesize that the involvement in epilepsy of NTFs, specifically BDNF and FGF-2, could go beyond their well-known effects on cell death, neurogenesis, and axonal sprouting [7], but also include the control of neuroinflammation.

Initial studies in cell cultures suggested that BDNF and FGF-2 may exert direct effects on the secretion of pro-inflammatory cytokines and, therefore, on neuroinflammation. However, data in this respect are inconclusive: (1) BDNF has been reported to decrease LPS-induced mRNA levels of TNF-α, IL-1β, and iNOS in astrocytes [31], but the question remains as to the actual levels of translation into proteins and secretion (in the case of TNF-α and IL-1β); (2) FGF-2 has been reported to decrease the release of TNF-α and IL-1β from astrocytes exposed to infrasound stimulation [32], but no concentration-response study has been performed, and it should be taken into account that infrasound is a very specific type of stimulation from which it is difficult to extrapolate general conclusions. In addition, the effect of combining the NTFs in vitro has never been studied. In this study, BDNF and FGF-2 were applied in an astrocyte–microglia mixed culture to further explore their mechanism of the modulation of neuroinflammation. No relevant effect could be detected at any concentration of FGF-2 and BDNF, alone or in combination, on LPS-induced cytokine release.

The most plausible interpretation of these negative data is that FGF-2 and BDNF do not act directly on the secretion of inflammatory factors by glial cells and, therefore, that the anti-inflammatory effects observed in vivo are indirect. For example, the attenuation of epilepsy-induced neuroinflammation [11] may be due to the reduced number of spontaneous seizures, which are responsible for chronic neuroinflammation. Needless to say, however, the in vivo situation of an epileptic tissue is far more complex than in the model systems employed in the present study. Therefore, the reductionist approach taken in this study cannot provide conclusive evidence in this respect, and further studies will be required to strengthen this hypothesis. Refined experimental designs should be used in the future to evaluate the effects of BDNF and FGF-2 at different time points of neuroinflammation development in vivo as well as in other cell culture compositions.

## 4. Materials and Methods

### 4.1. Chemicals and Antibodies

High-glucose Dulbecco’s Modified Eagle Medium (DMEM) w/L-Glutamine and sodium pyruvate (sterile filtered) were purchased from Carlo Erba, Italy. Streptomycin/penicillin cocktail (P0781), Lipopolysaccharide (LPS; Escherichia coli strain O111:B4; L4391), brain-derived neurotropic factor (BDNF; SRP3014), and fibroblast growth factor-2 (FGF-2; F0291), as well as Glial fibrillary acidic protein (GFAP; G9269), Triton-X100 (T8787), 4′,6-diamidino-2-phenylindole (DAPI; MBD0015), Fluoromount^TM^ aqueous mounting medium (F4680), bovine serum albumin (BSA; A3059), sulphanilamide (S9251), Hank’s balanced salt solution (HBSS; H-9394), L-Glutamine 200 mM (G7513), and paraformaldehyde (PFA; 158127), were obtained from Sigma-Aldrich (Merk Life Science S.r.l, Milan, Italy). Heat-inactivated fetal bovine serum (FBS; ECS0180L) and Trypsin-EDTA in phosphate-buffered saline (PBS; ECB3052D) were supplied by EuroClone (Euroclone S.p.A., Pero, Milan, Italy). PBS (pH 7.4; 10010-023), B-27™ supplement (17504-044l), Na^+^-pyruvate (11360-039), and neurobasal-A medium (21103) were from Gibco (Thermo Fisher Scientific, Watham, MA, USA). The microglial marker Ionized calcium binding adaptor molecule 1 (Iba1; 234003) was purchased from Synaptic Systems GmbH (Goettingen, Germany). Cross-adsorbed secondary antibody and Alexa Fluor™ 594 and 488 were acquired from Invitrogen (Watham, MA, USA) while naphthyl-ethylendiamine dihydrochloride (1062370025) was from Merck (Roma, Italy).

### 4.2. Primary Mixed Glial Culture and Neuronal Culture

The experiments were approved by the Italian Ministry of Health (authorization number: CBCC2.N.NUJ). Murine-derived primary MGCs were prepared from cerebral cortices of C57BL/6J mice (P0-1) according to Mecha (2011) [36] with slight modifications. Briefly, pups were decapitated, meninges and blood vessels were removed carefully under sterile conditions, and then cortices were dissected, mechanically dissociated via repeated pipetting in chilled high-glucose DMEM supplemented with 1% penicillin and 10 mg/mL streptomycin, and centrifuged at 1000 rpm for 10 min. The supernatant was removed and the pellet resuspended in pre-warmed high-glucose DMEM supplemented with the above-mentioned antibiotics and 10% heat-inactivated FBS.

Cells were cultured in 75 cm^2^ flask (VWR^®^, Radnor, PA, USA) and maintained at 37 °C with humidified atmosphere of 95% air/5% CO_2_. The medium was changed after 1 day in vitro (DIV) and then every 3 days. Once confluency was reached, attached cells were removed through trypsinization. The culture was characterized by immunocytochemistry using GFAP as specific astrocyte marker and Iba1 as microglial marker.

The 8 DIV cells were seeded in multi-well plates. To evaluate cell viability (MTT assay), cells were seeded in a 96-well plate, with 30,000 cells/well/100 μL. To evaluate NO and cytokine production, cells were seeded in a 48-well plate, with 60,000 cells/well/200 μL.

For neuronal culture, pups (P0-1) were decapitated, meninges and blood vessels were removed carefully under sterile conditions, and then cortices were dissected. The tissue was washed with HBSS and then incubated in 10% of trypsin in HBSS at 37 °C for 8–10 min for tissue digestion. The action of trypsin was blocked by adding 1 mL of Neurobasal medium containing 10% of FBS, 10 μL/mL Hepes, 10 μL/mL Na+-pyruvate, 1% penicillin, 10 mg/mL streptomycin, and 2.5 μL/mL L-glutamine. Following this, the tissue was homogenized through repeated pipetting using a Pasteur pipette and centrifuged at 500 rpm for 10 min. Then, the supernatant was removed and the pellet was resuspended in Neurobasal medium. Finally, the cells were filtered using a 70 μm nylon cell strainer. Cells were cultured in 75 cm^2^ flask (VWR^®^) and maintained at 37 °C with humidified atmosphere of 95% air/5% CO_2_. The medium was changed next day with fresh Neurobasal medium supplemented with 2% B-27™, 1% penicillin, 10 mg/mL streptomycin, and 2.5 μL/mL L-glutamine in the absence of FBS. Cells were allowed to reach confluency.

### 4.3. Treatments

At 8 DIV, cells were stimulated with different concentrations (1 to 10,000 ng/mL) of LPS for 24 h, 48 h, and 72 h in order to determine the concentration- and time-dependent effect of LPS on NO production and release of cytokines.

The effects of LPS on cell viability were measured 72 h post LPS treatment. To study the effects of BDNF and FGF-2 on cell viability, cytokine release, and NO production, cells were first treated with LPS 100 ng/mL or vehicle for 24 h and then the medium was replaced with a new one without LPS and with BDNF (0.1 to 1000 ng/mL) or FGF-2 (0.1 to 1000 ng/mL) or their combination in concentration ratios of 1:10 and 1:1 (FGF-2: BDNF) for 48 h. The incubation time and LPS concentration were chosen on the basis of the aforementioned experiments. During all experimental phases, cells were maintained in high-glucose DMEM supplemented with 1% penicillin, 10 mg/mL streptomycin, and 10% heat-inactivated FBS.

### 4.4. Immunocytochemistry

The cell phenotype was analyzed via performing immunocytochemistry experiments according to Li et al. (2016) [37] with slight modifications. The cells were seeded in 24-well culture plate with poly-L-lysine-coated coverslip and allowed 24 h incubation. They were then washed twice with PBS 1X and fixed with 4% PFA for 20 min at room temperature (RT). Cells were washed with PBS 1X to remove the residual fixative and incubated with 0.1% Triton-X100 (T8787-100ML; Sigma-Aldrich) for 10 min. After two rapid washes with PBS 1X, aspecific binding sites were blocked by using a blocking solution composed of 5% normal goat serum and 5% bovine serum albumin in PBS 1X for 60 min at RT. Overnight incubation with primary antibody anti-GFAP (1:200) or anti-Iba1 (1:500) was performed at 4 °C. On the following day, cells were washed again with PBS 1X and incubated with the respective cross-absorbed secondary antibodies (1:500) for 1 h at RT. Counterstaining with DAPI (1 μg/mL) was performed to visualize nuclei. Finally, cells were mounted with Fluoromount TM Aqueous Mounting Medium and images were acquired using a Leica DMRA2/RXA2 microscope (Leica Biosystems Nussloch GmbH, Nussloch (Germany)).

### 4.5. MTT Assay

Cell viability was assessed according to the instructions of manufacturer using a commercially available kit (Cell proliferation kit I MTT; Roche, Basel, Switzerland; 11465007001). Briefly, 10 μL MTT labelling reagent 1X was added in each well and incubated at 37 °C for 4 h. A quantity of 100 μL of solubilization buffer was then added in each well and incubated overnight at 37 °C. On the next day, the results were evaluated by measuring the absorbance at 550 nm using the multimode plate reader EnSight (PerkinElmer, Shelton, CT, USA).

### 4.6. Enzyme-Linked Immunosorbent Assay (ELISA)

The cell supernatant was collected and, in a first set of experiments, levels of a large set of cytokines were measured using the MILLIPLEX MAP Mouse Cytokine/Chemokine Multiplex Assay (Merck; MCYTOMAG-70K). Subsequent experiments focused on the subset of cytokines whose levels were increased by LPS (namely IL-1β, TNF-α, and IL-6). Their levels were measured using a commercially available simple plex 5th generation cartridge (Bio-Techne, Minneapolis, MN, USA; SPCKC-MP-005977) and the readings were taken using Ella automated immunoassay system (Bio-Techne) according to the manufacturer’s instructions. In addition, IL-4 and IL-10 were also measured using ELISA (catalog BSM614 and BMS613, respectively, from Thermo Fisher Scientific) and the multimodal plate reader Ensight (PerkinElmer).

### 4.7. Nitric Oxide Measurement

The production of NO was evaluated as the accumulated nitrate concentration in culture medium by using a Griess-reaction-based colorimetric reaction [13]. Briefly, 100 μL of a color reagent (0.1% naphthyl-ethylendiamine dihydrochloride in bidistilled water and 1% sulphanilamide in 5% orthophosphoric acid) was incubated with an equal volume of sample supernatant. After 5–10 min of incubation at RT, the absorbance was determined at 570 nm using the multimode plate reader EnSight (PerkinElmer). The standard calibration curve was generated using sodium nitrite standards (0–100 μM).

### 4.8. Statistical Analysis

Statistical analysis of data was performed using the Prism 6 software (GraphPad Inc., Boston, MA, USA). Data in this paper are shown as fold-change from the control, where control comprises vehicle-treated cells in LPS concentration-response curve experiments and LPS-treated cells in neurotrophic factor experiments. All results are means ± SEMs (standard errors of mean) of at least three independent experiments performed in duplicate. Data were analyzed using Kruskal–Wallis one-way ANOVA and post hoc Dunn’s test. Data were considered statistically significant when *p* < 0.05.

## Data Availability

The data that support the findings of this study are available from the corresponding author, C.R., upon request.
